# Adjunct prednisone in community-acquired pneumonia: 180-day outcome of a multicentre, double-blind, randomized, placebo-controlled trial

**DOI:** 10.1186/s12890-023-02794-w

**Published:** 2023-12-11

**Authors:** Claudine A. Blum, Eva A. Roethlisberger, Nicole Cesana-Nigro, Bettina Winzeler, Nicolas Rodondi, Manuel R. Blum, Matthias Briel, Beat Mueller, Mirjam Christ-Crain, Philipp Schuetz

**Affiliations:** 1 Endocrinology, Diabetology and Metabolism, Department of Internal Medicine and Department of Clinical Research, University Hospital Basel, 4031 Basel, Switzerland; 2 Medical University Clinic, Division of General Internal & Emergency Medicine and Division of Endocrinology, Diabetology and Metabolism, Kantonsspital Aarau, 5001 Aarau, Switzerland; 3 Hormonpraxis Aarau, 5000 Aarau, Switzerland; 4 Stoffwechselzentrum, Bürgerspital, 4500 Solothurn, Switzerland; 5 Institute of Primary Health Care (BIHAM), University of Bern, Bern, Switzerland; 6 Department of General Internal Medicine, Inselspital, Bern University Hospital, University of Bern, 3010 Bern, Switzerland; 7 CLEAR-Methods Center, Division of Clinical Epidemiology, Department of Clinical Research, University Hospital Basel and University of Basel, 4031 Basel, Switzerland; 8 Department of Health Research Methods, Evidence, and Impact, McMaster University, Hamilton, Ontario Canada

**Keywords:** Community-acquired pneumonia, Corticosteroids, Glucocorticoids, Prednisone, Lower respiratory tract infections

## Abstract

**Background:**

Several trials and meta-analyses found a benefit of adjunct corticosteroids for community-acquired pneumonia with respect to short-term outcome, but there is uncertainty about longer-term health effects. Herein, we evaluated clinical outcomes at long term in patients participating in the STEP trial (Corticosteroid Treatment for Community-Acquired Pneumonia).

**Methods:**

This predefined secondary analysis investigated 180-day outcomes in 785 adult patients hospitalized with community-acquired pneumonia included in STEP, a randomised, placebo-controlled, double-blind trial. The primary endpoint was time to death from any cause at 180 days verified by telephone interview. Additional secondary endpoints included pneumonia-related death, readmission, recurrent pneumonia, secondary infections, new hypertension, and new insulin dependence.

**Results:**

From the originally included 785 patients, 727 were available for intention-to-treat analysis at day 180. There was no difference between groups with respect to time to death from any cause (HR for corticosteroid use 1.15, 95% CI 0.68 to 1.95, *p* = 0.601). Compared to placebo, corticosteroid-treated patients had significantly higher risks for recurrent pneumonia (OR 2.57, 95% CI 1.29 to 5.12, *p* = 0.007), secondary infections (OR 1.94, 95% CI 1.25 to 3.03, *p* = 0.003) and new insulin dependence (OR 8.73, 95% CI 1.10 to 69.62, *p* = 0.041). There was no difference regarding pneumonia-related death, readmission and new hypertension.

**Conclusions:**

In patients with community-acquired pneumonia, corticosteroid use was associated with an increased risk for recurrent pneumonia, secondary infections and new insulin dependence at 180 days. Currently, it is uncertain whether these long-term adverse effects outweigh the short-term effects of corticosteroids in moderate CAP.

**Trial registration:**

This trial was registered with ClinicalTrials. gov, number NCT00973154 before the recruitment of the first patient. First posted: September 9, 2009. Last update posted: April 21, 2015.

**Supplementary Information:**

The online version contains supplementary material available at 10.1186/s12890-023-02794-w.

## Background

Lower respiratory tract infections such as pneumonia are the most deadly infectious disease worldwide and the world’s fourth common cause of death, according to WHO publication in December 2020 [1]. In spite of adequate antimicrobial treatment in community-acquired pneumonia (CAP), the mortality and morbidity remain substantial [2]. An excessive inflammatory systemic cytokine release in CAP [3], as well as cytokine storm in COVID-19 pneumonitis can have detrimental impact on the lung [4]. Herein, anti-inflammatory effects of systemic corticosteroids attenuate this harmful process and thus may improve clinical outcomes of patients [3]. In the past, several randomized controlled trials and two recent meta-analyses investigated adjunct corticosteroids in treatment of CAP. Adjunct corticosteroids in CAP shorten time to clinical stability and duration of hospital stay, whereas a mortality benefit was only shown for severe CAP [5–7]. However, corticosteroids also have well known side effects including in-hospital hyperglycaemia [5, 6]. Also, the meta-analysis by Briel et al. showed an increase of CAP-related rehospitalisation [6].

The Infectious Disease Society of America (IDSA)/American Thoracic Society (ATS) guidelines published in 2019 recommended supplementary treatment with corticosteroids in treatment of septic shock in CAP [8]. The investigated period of time was approximately 30 days, but one analysis included a study with 60 days follow-up [9]. In the COVID-19 pandemic, the RECOVERY trial dexamethasone improved short-term outcome in severe COVID-19 pneumonitis by 20–30% [10].

These findings support short-term corticosteroid administration in order to prevent pulmonary dysfunction induced by hyperinflammation, for both, viral COVID-19-pneumonitis and in bacterial community acquired pneumonia, even in moderate cases hospitalized outside the ICU [11].

However, whether corticosteroids have a lasting effect on outcome beyond 30 days needs further exploration.

We herein analysed the 180 day - outcome of patients included into the STEP-trial [12], which investigated 50 mg prednisone vs. placebo in about 800 patients hospitalized with CAP. The original trial showed a reduction of time to clinical stability and duration of hospital stay, without an increase in complications. In addition, a secondary analysis of the initial STEP trial indicated favourable effects of corticosteroids despite of pre-existing diabetes or hyperglycaemia due to adjunct corticosteroids [13].

## Methods

### Study setting and participants

This is a pre-planned secondary analysis of a multicentre, double-blind, randomised, placebo-controlled trial, which investigated adjunct prednisone for patients with CAP on the primary outcome of time to death from any cause. The study protocol of the initial STEP-trial has been published elsewhere [14]. In brief, consecutive patients presenting with community-acquired pneumonia were screened and enrolled at emergency departments or medical wards in seven tertiary care hospitals in Switzerland from Dec 1, 2009, to May 21, 2014, within 24 h of presentation. Inclusion criteria were age 18 years or older and hospital admission with community-acquired pneumonia defined by a new infiltrate on chest radiograph and the presence of at least one of the following acute respiratory signs and symptoms: cough, sputum production, dyspnoea, core body temperature of 38·0 °C or higher, auscultatory findings of abnormal breathing sounds or rales, leucocyte count higher than 10,000 cells per μL or less than 4000 cells per μL [15]. Exclusion criteria were permanent inability for informed consent, active intravenous drug use, acute burn injury, gastrointestinal bleeding within the past 3 months, known adrenal insufficiency, a condition requiring more than 0.5 mg/kg per day prednisone equivalent, pregnancy or breastfeeding, and severe immunosuppression defined as one of the following: infection with human immunodeficiency virus and a CD4 cell count below 350 cells per μL, immunosuppressive therapy after solid organ transplantation, neutropenia below 500 cells per μL or neutrophils of 500–1000 cells per μL during ongoing chemotherapy with an expected decrease to values below 500 cells per μL, cystic fibrosis, or active tuberculosis. The conduct of the trial adhered to the declaration of Helsinki and Good Clinical Practice Guidelines, and ethical committees of all participating hospitals approved the study before patient recruitment. All patients provided written informed consent [14]. This trial was registered with ClinicalTrials. gov, number NCT00973154 before the recruitment of the first patient. First posted: September 9, 2009. Last update posted: April 21, 2015 [14].

### Randomization and blinding

Eligible patients were randomly assigned (1:1 ratio) to receive either 50 mg of prednisone or placebo daily for 7 days. Randomisation was done with variable block sizes of four to six and patients were stratified at the time of study entry by study centre. Allocation was concealed with a prespecified computer-generated randomisation list, which was centrally kept at the pharmacy of the main study centre. Patients were randomly assigned to receive a prepared set of study medication that contained seven tablets of 50 mg prednisone or placebo. The placebo drug was purchased from a local prednisone manufacturer (Galepharm AG, Küsnacht, Switzerland), which produces both prednisone and its corresponding placebo. The drugs were prepared before the initiation of the study and packed into identical containers by the Pharmacology Department, University Hospital, Basel, according to the randomisation list. Patients, treating physicians, investigators, and data assessors were blinded to treatment allocation until day 30 [14].

### Procedures

After informed consent was obtained, baseline blood samples were drawn and nasal swabs for virus multiplex PCR were done. All other microbiological assessments were at the discretion of the treating physicians. Patients started antibiotic therapy as soon as community acquired pneumonia was confirmed. Treating physicians chose the empirical regimen according to the ERS/ ESCMID guidelines adapted for Switzerland [16, 17]. Most patients started this regimen either with amoxicillin plus clavulanic acid or ceftriaxone alone. In patients with clinical suspicion for legionellosis or in those requiring treatment in the intensive care unit (ICU), the betalactam was combined with clarithromycin. Treatment was streamlined and optimised according to the susceptibility pattern as soon as a specific pathogen was known. Thereafter, patients started receiving study medication, and we monitored timing in relation to start of antibiotics. Study nurses assessed patients for clinical stability every 12 h during hospital stay. All patients were treated according to published community-acquired pneumonia guidelines [18]. Stewardship of antibiotic treatment duration by procalcitonin was encouraged by the study protocol according to Schuetz and colleagues [19]. Baseline data included medical history items, relevant comorbidities, clinical items of pneumonia, and all variables required for the calculation of the pneumonia severity index (PSI) [20]. Routine laboratory tests of inflammatory markers (procalcitonin, C-reactive protein [CRP], white blood cell count) were done in both groups on days 1, 3, 5, 7, and before discharge and included four glucose measurements per day. Structured follow-up telephone interviews for secondary outcomes after discharge were done on day 30 and on day 180. The interview included the assessment of adverse events such as secondary infections, recurrent pneumonia, re-admission to hospital, new insulin dependence, new onset hypertension, and mortality, which was further classified if CAP-related or not [14]. All items of the follow-up assessment, including outcome, were assessed by telephone interview with the patient, or, if unavailable, with next of kin or the responsible general practitioner. In cases with re-admission, outcome measures were cross-checked with the electronic patient chart at the hospital.

### Outcomes und endpoints

The objective of this analysis was to investigate differences in outcome until 180 days after adjunct prednisone or placebo in CAP. Specifically, we analysed the two treatment groups for differences in the following primary and secondary endpoints. Furthermore, subgroup analysis for predefined endpoints was performed.

The primary endpoint was defined as time to death from any cause.

Secondary endpoints included CAP-related death, readmission, recurrent pneumonia, secondary infections, new hypertension at day 180 and new insulin dependence at day 180.

Types of secondary infections were categorized as dermatological, urogenital, pulmonary, intestinal, and endocardium/ foreign body infections, as well as a group of combined urogenital and pulmonary infections. The last group was formed due to the frequent issue of the two infections often occurring simultaneously.

In the original trial protocol, the primary outcome was time to clinical stability. Secondary endpoints in the original protocol were all-cause mortality, time to effective hospital discharge, and side effects of corticosteroid treatments, among others [12].

### Statistical analysis

The statistical analysis was prespecified, and investigators who analysed the data were masked to treatment allocation [14]. The primary hypothesis was that adjunct prednisone in community-acquired pneumonia does not increase mortality or other adverse events up to 180 days. The analysis was performed with the 180-day data of the intention-to-treat population and therefore focused on patients included in the trial until day 180. For the primary endpoint, we calculated an adjusted hazard ratio (HR) and 95% CI using Cox proportional hazards regression based on the binary outcome of survival or death from any cause. Secondary endpoints were calculated by logistic regression based on the binary outcome of occurrence of particular adverse event or no occurrence.

As a sensitivity analysis, the same analyses were performed in the per-protocol population.

Results are given as adjusted odds ratio (OR) and with 95% CI. In primary and secondary outcomes, hazard ratio and odds ratio were adjusted for PSI and age.

The prespecified subgroup analyses [14] (patient age, CRP concentration on admission, history of chronic obstructive pulmonary disease [COPD], PSI class, blood culture positivity) were conducted by including appropriate interaction terms in the multivariable Cox proportional hazards model or logistic model as appropriate [14]. All reported CIs are two-sided 95% intervals, and tests were done at the two-sided 5% significance level. We used STATA 15.0 (Stata Corp, College Station, Texas) for all analyses.

## Results

### Patient population

**Fig. 1:**
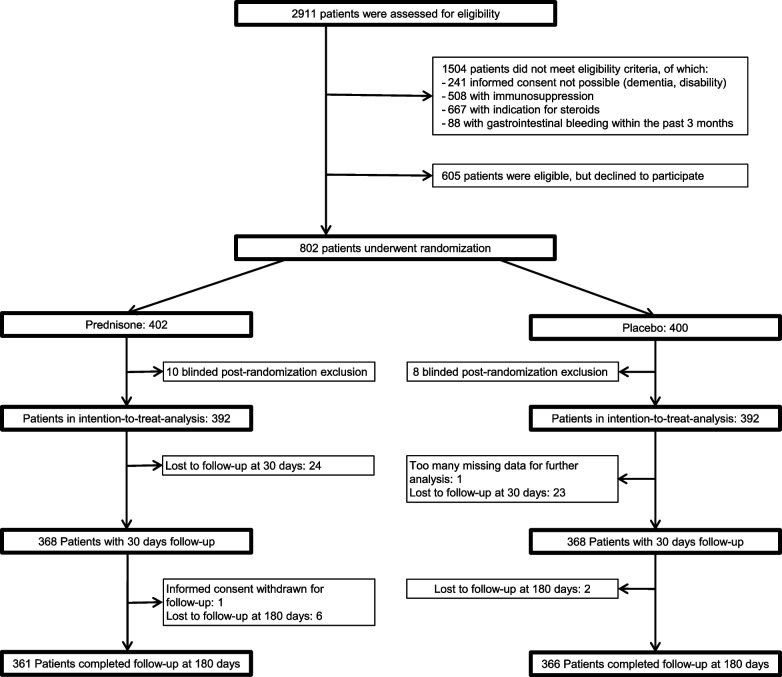
Trial flow chart

Eight hundred two eligible patients were enrolled in the original trial and randomly assigned to receive either prednisone or placebo (see Fig. 1).

The difference between study flow chart of STEP analysis 30 days and 180 days is due to new information and change of diagnosis during hospitalisation (one patient suffered of diverticulitis instead of pneumonia).

After blinded post-randomisation exclusion of 18 patients not meeting eligibility criteria, 392 patients were allocated to the prednisone group and 392 patients to the placebo group. The intention-to-treat analysis further excluded 57 patients (i.e., 47 patients were lost to follow-up at 30 days, eight patients were lost to follow-up at 180 days, one patient withdrew informed consent to be contacted at 180 days, and one patient had too many missing data for this secondary analysis). Therefore, 361 patients were available for the intention-to-treat analysis at 180 days in the prednisone arm and 366 patients in the placebo arm.

Baseline characteristics between the two arms were balanced (see Table 1).

**Table 1:** Baseline characteristics of enrolled patients

	Placebo (*n* = 366)	Prednisone (*n* = 361)
**General characteristics**		
Age, median (IQR), y	73 (61, 82)	74 (61, 84)
Male Gender, No. (%)	231 (63.1%)	219 (60.7%)
**Clinical variables**		
Days with symptoms, median (IQR), d	4 (2, 7)	4 (2, 7)
Temperature, median (IQR), °C	37.6 (37, 38.3)	37.5 (37, 38.2)
Systolic blood pressure, median (IQR), mmHg	124 (110, 140)	124 (110, 140)
Heart rate, median (IQR), bpm	82 (71, 96)	84 (74, 94)
Respiratory Rate, median (IQR), bpm	20 (18, 24)	20 (18, 24)
SaO2, median (IQR), %	94 (92, 96)	94.5 (92, 96)
Confusion, No. (%)	27 (7.4%)	21 (5.8%)
**Laboratory values**		
Procalcitonin, median (IQR), ng/mL	0.43 (0.17, 2.3)	0.46 (0.17, 2.53)
C-reactive protein, median (IQR), mg/L	159 (73.8, 249)	156 (80, 245.5)
Leukocytes, median (IQR), × 10^9^/L	11.98 (8.78, 15.6)	12.1 (8.88, 15.5)
**Microbiological data**		
Bacteraemia, No. (%)	45 (12.3%)	37 (10.2%)
Bacterial etiology confirmed	87 (23.8%)	81 (22.4%)
Viral etiology confirmed	38 (17.4%)	38 (17.8%)
**PSI score**^a^		
PSI class I, No. (%)	41 (11.2%)	44 (12.2%)
PSI class II, No. (%)	66 (18.0%)	66 (18.3%)
PSI class III, No. (%)	86 (23.5%)	64 (17.7%)
PSI class IV, No. (%)	125 (34.2%)	137 (38.0%)
PSI class V, No. (%)	48 (13.1%)	50 (13.9%)
Total PSI score, median (IQR),points	86 (65, 110)	93 (63, 115)
**Comorbidities**		
Diabetes mellitus, No. (%)	72 (19.7%)	66 (18.3%)
Insulin treatment, No. (%)	23 (32%)	23 (35%)
Chronic obstructive pulmonary disease, No. (%)	54 (14.8%)	70 (19.4%)
Heart failure, No. (%)	58 (15.9%)	74 (20.5%)
Cerebrovascular disease, No. (%)	30 (8.2%)	33 (9.1%)
Renal insufficiency, No. (%)	117 (32.1%)	116 (32.1%)
Neoplasia, No. (%)	22 (6.0%)	27 (7.5%)
Liver disease, No. (%)	11 (3.0%)	17 (4.7%)
Antibiotic pretreatment, No. (%)	89 (24.3%)	76 (21.1%)

Data are median (IQR) or number (%), unless otherwise stated. *SaO2* saturation of oxygen. *PSI* pneumonia severity index

^a^The PSI is a clinical prediction rule to calculate the probability of morbidity and mortality in patients with community-acquired pneumonia; PSI risk class I corresponds to age ≤ 50 years, and no risk factors (≤50 points), risk class II to < 70 points, risk class III to 71–90 points, risk class IV to 91–130 points, and risk class V to > 130 points [20]

Median age was 73 years in the placebo group and 74 years in the prednisone group. 63.1% were men in the placebo group and 60.7% in the prednisone group. Patients had a high burden of comorbidities including diabetes, chronic obstructive pulmonary disease, chronic heart failure, and chronic renal insufficiency. About half the patients were in high-risk PSI classes IV and V.

### Main outcome results

**Fig. 2:**
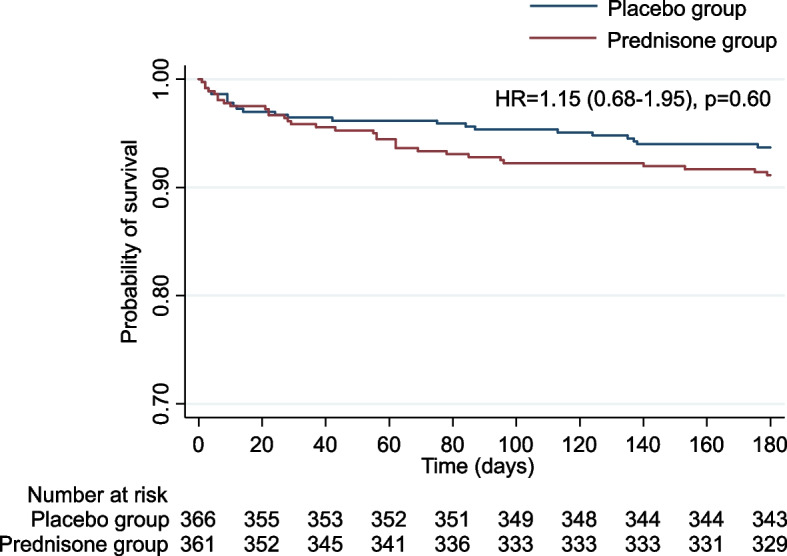
Kaplan-Meier-curve of time to death from any cause

The main results for the primary and secondary endpoints are shown in Table 2 and Fig. 2.

**Table 2:** Overview of primary and secondary endpoints

Endpoints	Placebo (*n* = 366)	Prednisone (*n* = 361)	Adjusted HR or OR (95%CI)^a^	*P* value
**Primary endpoint**				
Death from any cause – no. (%)	25 (6.8%)	35 (9.7%)	HR 1.15 (0.68–1.95)	0.601
**Secondary endpoints**				
CAP-related death	7 (1.9%)	6 (1.7%)	OR 0.75 (0.24–2.33)	0.624
Re-Hospitalization – no. (%)	55 (15.0%)	70 (19.4%)	OR 1.33 (0.90–1.96)	0.158
Reason for re-hospitalization – no. (%)				
- Recurrent pneumonia	5 (9%)	21 (30%)		
- Other infection	3 (5.5%)	4 (5.7%)		
- other	41 (74.5%)	39 (55.7%)		
- not reported	6 (11%)	6 (8.6%)		
Recurrent pneumonia – no. (%)	12 (3.3%)	29 (8.0%)	OR 2.57 (1.29–5.12)	0.007
Secondary infections	35 (9.6%)	62 (17.2%)	OR 1.94 (1.25–3.03)	0.003
Type of infection				
- dermatological	1 (3%)	5 (8%)		
- urogenital	10 (29%)	9 (15%)		
- pulmonary	12 (35%)	18 (30%)		
- intestinal	10 (29%)	22 (37%)		
- endocardium or foreign body	1 (3%)	4 (7%)		
- both urogenital and pulmonary	0 (0%)	2 (3%)		
Empyema	7 (1.9%)	3 (0.8%)	OR 0.44 (0.11–1.73)	0.242
New hypertension at day 180	6 (1.6%)	11 (3.0%)	OR 1.90 (0.69–5.18)	0.213
New insulin dependence at day 180	1 (0.3%)	9 (2.5%)	OR 8.73 (1.10–69.62)	0.041

Data are number (%) unless otherwise stated. *HR* hazard ratio. *OR* odds ratio. *CI* confidence interval. *CAP* community-acquired pneumonia

^a^Adjusted for PSI^#^ and age

^#^*PSI* pneumonia severity index. The PSI is a clinical prediction rule to calculate the probability of morbidity and mortality in patients with community-acquired pneumonia; PSI risk class I corresponds to age ≤ 50 years, and no risk factors (≤50 points), risk class II to < 70 points, risk class III to 71–90 points, risk class IV to 91–130 points, and risk class V to > 130 points [20]

There was no difference between prednisone and placebo groups regarding the time to death from any cause over the 180 day follow-up (HR 1.15, 95% CI 0.68–1.95, *p* = 0.601). Similarly, CAP-related death was not different between treatment groups (OR 0.75, 95% CI 0.24–2.33, *p* = 0.624). This was also true for hospital readmission (OR 1.33, 95% CI 0.90–1.96, *p* = 0.158) and new hypertension at day 180 (OR 1.90, 95% CI 0.69–5.18, *p* = 0.213). However, recurrent pneumonia (OR 2.57, 95% CI 1.29–5.12, *p* = 0.007), secondary infections (OR 1.94, 95% CI 1.25–3.03, *p* = 0.003), and new insulin dependence at day 180 (OR 8.73, 95% CI 1.10–69.62, *p* = 0.041) occurred more often in the prednisone group compared to the control group.

Disease activity scores were similar between treatment groups at day 180 (see supplemental Table 6).

### Subgroup analysis

We performed several predefined subgroup analyses for the primary endpoint mortality and for the secondary endpoints recurrent pneumonia as well as secondary infection.

We noted no evidence of effect modification in different prespecified subgroups based on median age, initial median CRP concentration, previous history of COPD, severity of community-acquired pneumonia defined by the PSI score (I–III vs IV–V). (see Tables 3, 4, and 5).

**Table 3:** Subgroup analyses for the primary endpoint mortality

Subgroup variables	Placebo (*n* = 366)	Prednisone (*n* = 361)	Cox regression, HR (95%CI)	*p* for heterogeneity (interaction)^b^
**Median age**				0.326
Age ≤ 73 years	8/192 (4.2%)	6/177 (3.4%)	0.97 (0.33–2.90)	
Age > 73 years	17/174 (9.8%)	29/184 (15.8%)	1.36 (0.74–2.51)	
**Initial median CRP**				0.801
CRP ≤ 158 mg/L	14/181 (7.7%)	19/180 (10.6%)	1.37 (0.67–2.78)	
CRP > 158 mg/L	11/182 (6.0%)	15/179 (8.4%)	1.10 (0.49–2.46)	
**History of Chronic Obstructive Pulmonary Disease**				0.989
no	21/312 (6.7%)	25/291 (8.6%)	1.26 (0.70–2.27)	
yes	4/54 (7%)	10/70 (14%)	1.51 (0.45–5.12)	
**Pneumonia severity index**^a^				0.632
PSI class I-III	4/193 (2.1%)	6/174 (3.4%)	1.77 (0.50–6.29)	
PSI class IV-V	21/173 (12.1%)	29/187 (15.5%)	1.14 (0.65–2.03)	
**Blood culture positivity**				0.505
Blood culture negative	22/321 (6.9%)	30/324 (9.3%)	1.12 (0.64–1.97)	
Blood culture positive	3/45 (7%)	5/37 (14%)	1.17 (0.24–5.64)	
**Sex**				0.445
Male	7/135 (5.2%)	16/142 (11.3%)	1.85 (0.75–4.60)	
Female	18/231 (7.8%)	19/219 (8.7%)	0.96 (0.49–1.88)	
**Microbiological etiology**				
Bacterial proof				0.429
Yes	5/87 (6%)	10/81 (12%)	1.83 (0.58–5.74)	
No	17/245 (6.9%)	22/254 (8.7%)	1.05 (0.55–2.02)	
Viral proof				0.364
Yes	1/38 (3%)	3/38 (8%)	0.44 (0.22–9.01)	0.364
No	16/181 (8.8%)	18/176 (10.2%)	1.05 (0.52–2.11)	

Data are number (%) unless otherwise stated. *HR* hazard ratio. *CI* confidence interval.

^a^The PSI is a clinical prediction rule to calculate the probability of morbidity and mortality in patients with community-acquired pneumonia; PSI risk class I corresponds to age ≤ 50 years, and no risk factors (≤50 points), risk class II to < 70 points, risk class III to 71–90 points, risk class IV to 91–130 points, and risk class V to > 130 points [20].

^b^Cox proportional hazards model including an interaction term of the respective subgroup variable with treatment group

**Table 4:** Subgroup analyses for the secondary endpoint recurrence

Subgroup variables	Placebo (*n* = 366)	Prednisone (*n* = 361)	Logistic regression, OR (95%CI)	*p* for heterogeneity (interaction)^b^
**Median age**				0.498
Age ≤ 73 years	8/192 (4.2%)	15/177 (8.5%)	2.21 (0.91–5.39)	
Age > 73 years	4/174 (2.3%)	14/184 (7.6%)	3.49 (1.12–10.84)	
**Initial median CRP**				0.251
CRP ≤ 158 mg/L	3/181 (1.7%)	13/180 (7.2%)	4.54 (1.27–16.23)	
CRP > 158 mg/L	9/182 (4.9%)	16/179 (8.9%)	1.89 (0.81–4.40)	
**History of Chronic Obstructive Pulmonary Disease**				0.991
no	9/312 (2.9%)	20/291 (6.9%)	2.49 (1.11–5.55)	
yes	3/54 (6%)	9/70 (13%)	2.58 (0.64–10.41)	
**Pneumonia severity index**^a^				0.869
PSI class I-III	6/193 (3.1%)	14/174 (8.0%)	2.75 (1.03–7.32)	
PSI class IV-V	6/173 (3.5%)	15/187 (8.0%)	2.54 (0.96–6.74)	
**Blood culture positivity**				0.014
Blood culture negative	7/321 (2.2%)	28/324 (8.6%)	4.23 (1.82–9.84)	
Blood culture positive	5/45 (11%)	1/37 (3%)	0.14 (0.01–1.75)	
**Sex**				0.730
Male	8/231 (3.5%)	17/219 (7.8%)	2.33 (0.98–5.53)	
Female	4/135 (3.0%)	12/142 (8.5%)	3.03 (0.95–9.66)	
**Microbiological etiology**				
Bacterial proof				0.013
Yes	7/87 (8%)	5/81 (6%)	0.71 (0.21–2.37)	
No	4/245 (1.6%)	22/254 (8.7%)	5.69 (1.93–16.77)	
Viral prof				0.200
Yes	3/38 (8%)	4/38 (11%)	1.57 (0.32–7.79)	
No	3/181 (1.7%)	14/176 (8.0%)	5.15 (1.45–18.29)	

Data are number (%) unless otherwise stated. *OR* odds ratio. *CI* confidence interval.

^a^The PSI is a clinical prediction rule to calculate the probability of morbidity and mortality in patients with community-acquired pneumonia; PSI risk class I corresponds to age ≤ 50 years, and no risk factors (≤50 points), risk class II to < 70 points, risk class III to 71–90 points, risk class IV to 91–130 points, and risk class V to > 130 points [20].

^b^Logistic regression including an interaction term of the respective subgroup variable with treatment group

**Table 5:** Subgroup analyses for the secondary endpoint secondary infection

Subgroup variables	Placebo (*n* = 366)	Prednisone (*n* = 361)	Logistic regression, OR (95%CI)	*p* for heterogeneity (interaction)^b^
**Median age**				0.328
Age ≤ 73 years	14/192 (7.3%)	29/177 (16.4%)	2.52 (1.28–4.95)	
Age > 73 years	21/174 (12.1%)	33/184 (17.9%)	1.60 (0.89–2.91)	
**Initial median CRP**				0.114
CRP ≤ 158 mg/L	18/181 (9.9%)	41/180 (22.8%)	2.65 (1.46–4.83)	
CRP > 158 mg/L	17/182 (9.3%)	21/179 (11.7%)	1.28 (0.65–2.52)	
**History of Chronic Obstructive Pulmonary Disease**				0.416
no	29/312 (9.3%)	44/291 (15.1%)	1.74 (1.06–2.86)	
yes	6/54 (11%)	18/70 (26%)	2.60 (0.94–7.20)	
**Pneumonia severity index**^a^				0.522
PSI class I-III	15/193 (7.8%)	28/174 (16.1%)	2.29 (1.18–4.44)	
PSI class IV-V	20/173 (11.6%)	34/187 (18.2%)	1.69 (0.93–3.08)	
**Blood culture positivity**				0.720
Blood culture negative	29/321 (9.0%)	52/324 (16.0%)	1.92 (1.19–3.12)	
Blood culture positive	6/45 (13%)	10/37 (27%)	2.11 (0.66–6.72)	
**Sex**				0.547
Male	19/231 (8.2%)	36/219 (16.4%)	2.20 (1.22–3.97)	
Female	16/135 (11.9%)	26/142 (18.3%)	1.66 (0.84–3.27)	
**Microbiological etiology**				
Bacterial proof				0.391
Yes	12/87 (14%)	17/81 (21%)	1.58 (0.70–3.57)	
No	19/245 (7.8%)	45/254 (17.7%)	2.57 (1.46–4.55)	
Viral proof				0.209
Yes	2/38 (5%)	9/38 (24%)	5.71 (1.12–29.10)	
No	20/181 (11.0%)	33/176 (18.8%)	1.85 (1.01–3.37)	

Data are number (%) unless otherwise stated. *OR* odds ratio. *CI* confidence interval.

^a^The PSI is a clinical prediction rule to calculate the probability of morbidity and mortality in patients with community-acquired pneumonia; PSI risk class I corresponds to age ≤ 50 years, and no risk factors (≤50 points), risk class II to < 70 points, risk class III to 71–90 points, risk class IV to 91–130 points, and risk class V to > 130 points [20].

^b^Logistic regression including an interaction term of the respective subgroup variable with treatment group

However, a significant subgroup effect was found in regard to recurrent pneumonia in the subgroup of patients with negative blood cultures, and in the subgroup of patients without bacterial proof, but not in the subgroup with viral proof.

The sensitivity analysis in the per-protocol population showed similar results (see Additional file 1 Supplemental Tables S1 to S5 and Supplemental Figs. F1 and F2).

## Discussion

In this 180-day analysis of a large randomized controlled trial in patients hospitalized with CAP, we found similar risks for mortality and most other secondary endpoints, but higher incidences of secondary infections, recurrent pneumonias and new insulin dependence in patients randomized to the active corticosteroid group.

While corticosteroids have shown beneficial effects at short term in the population of patients with CAP including shorter time to stabilization and lower mortality [5–7, 12], longer-term data have been scarce and have suggested possible harm [21–23]. Herein, the recurrence of CAP and other secondary infections in the corticosteroid group are in line with previous research and may be explained by a blunting of the systemic immune reaction and a roughly 30% higher rate of hyperglycemia, increasing the risk of infections [13, 24].

There is limited availability of evidence on outcome after CAP beyond day 30 [25, 26]. Most studies investigated more long-term mortality around 5 years and show an increased mortality rate after CAP. Risk factors for mortality beyond day 30 include initial CAP severity, comorbidities, initially high inflammatory markers, and nursing home residency, apart from age [25–28]. Concerning adjunct corticosteroids, most trials did not do a follow-up beyond day 30. The available meta-analyses on corticosteroids in CAP do not indicate an increase of secondary infections, but this evidence does not go beyond the 30 day- follow-up [29, 30]. However, studies investigating adverse effects after short-term prescription of corticosteroids have shown an increase of secondary infections even beyond day 30 [21–23].

Whereas the absolute number of new insulin dependence at day 180 was low, it was significantly higher in the prednisone group. Various individual risk factors for the development of corticosteroid-induced diabetes have been identified [31]. Before administering corticosteroids, this risk should be evaluated and hyperglycemia should be treated to prevent further adverse events [32].

We performed subgroup analyses with the aim to identify specific subgroups which would be more susceptible to adverse events, but we could not assert any effect modification by median age, initial median CRP, previous history of COPD, or severity of community-acquired pneumonia. We did find that patients without bacteremia and without proof of bacterial infection had a higher incidence of recurrence of pneumonia when receiving corticosteroids, but there was no effect modification by viral etiology of pneumonia. One might speculate that this could be a subset of patients which did, in fact, not have infectious pneumonia but rather a pneumonitis, which was overseen as generally considered uncommon in the pre-COVID-19-era.

In another subanalysis with 30-day data, a phenotype with high cytokines benefited most from adjunct corticosteroids, as published elsewhere [33, 34].

The meta-analysis by Stern et al. showed a mortality benefit only in patients with severe CAP [5]. As our trial included a high number of patients with CAP of moderate severity, these patients benefited from a shorter length of stay and shorter duration of intravenous antibiotic treatment, but, overall, potentially had a negative effect concerning more CAP recurrence and secondary infections beyond day 30. Therefore, the question stands out whether these shorter-term benefits of corticosteroids in non-critically ill patients with CAP outweigh the late adverse effects. The following factors should be considered: First, a shorter length of stay has obvious socio-economic advantages, such as lower costs, lower usage of hospital infrastructure and less work load for the hospital staff [35–37]. Second, a shorter length of stay may be considered one of the preventive measures to reduce nosocomial infections. Third, in frail elderly patients, hospitalization commonly leads to immobilization, which may lead to functional decline and an increased risk for falls, delirium and other adverse events [38, 39]. In the frail elderly subgroup, a reduction of length of stay means also a potential reduction of these adverse events. Fourth, a shorter duration of intravenous antibiotic treatment is one of the measures to prevent secondary infections due to phlebitis [40].

In our trial, secondary infections during hospital stay and at 30 days were not increased in the prednisone group. The reduction in length of stay at day 30 may have outbalanced the increased risk for secondary infections conferred by the corticosteroids.

In summary, when considering adjunct corticosteroids for CAP, it is necessary to weigh the anticipated benefits like faster time to clinical stability, shorter length of stay and shorter duration of intravenous antibiotic treatment against potential adverse effects like in-hospital hyperglycemia, secondary infections beyond day 30 and recurrence of CAP beyond day 30.

### Limitations

The major limitation of this analysis is the question whether short-term corticosteroids are still causative for the effects found at day 180. The trial was not powered for these secondary endpoints; therefore, the results should be considered hypothesis generating.

In addition, despite standardized questions in the endpoints, there is a risk of information bias due to the assessment of data as patient interviews.

It must be acknowledged that COVID-19 pneumonitis and CAP are different diseases and thus comparison is limited, in spite of similarities in subsequent hyperinflammation and pulmonary affection.

The main strengths of the trial are the large number of patients and the randomized placebo-controlled design, preventing many confounders. Moreover, this is the first randomized controlled trial of corticosteroids in CAP investigating long-term outcomes until 180 days.

## Conclusion

In patients with community-acquired pneumonia, corticosteroid use was associated with an increased risk for recurrent pneumonia, secondary infections, and new insulin dependence in the following 180 days. Currently, it is uncertain whether these long-term adverse effects outweigh the short-term effects of corticosteroids in moderate CAP.

### Supplementary Information

**Additional file 1: Supplemental Table S1. **Baseline characteristics of per-protocol population. **Supplemental Table S2.** Overview of primary and secondary endpoints per-protocol. **Supplemental Table S3.** Subgroup analyses per-protocol for the primary endpoint mortality. **Supplemental Table S4.** Subgroup analyses per-protocol for the secondary endpoint recurrence. **Supplemental Table S5.** Subgroup analyses per-protocol for the secondary endpoint secondary infection. **Supplemental Table S6.** Overview of disease activity scores of intention-to-treat population. **Supplemental Figure F1.** Trial flow chart per-protocol. **Supplemental Figure F2.** Kaplan-Meier-curve of time to death per-protocol.

## Data Availability

The datasets analysed during the current study are available from the corresponding author on reasonable request.

## Abbreviations

CAP: community-acquired pneumonia
COPD: Chronic Obstructive Pulmonary Disease
CRP: C-reactive protein
ERS: European Respiratory Society
ESCMID: European Society of Clinical Microbiology and Infectious Diseases
ICU: Intensive Care Unit
PCT: Procalcitonin
PSI: pneumonia severity index
