# Supplementary material for: Adjunct prednisone in community-acquired pneumonia: 180-day outcome of a multicentre, double-blind, randomized, placebo-controlled trial
Source: BMC Pulm Med. 2023 Dec 11;23:500. doi: 10.1186/s12890-023-02794-w (PMC10712075; doi:10.1186/s12890-023-02794-w)
Supplement: Supplementary file 1 — Additional file 1: Supplemental Table S1. Baseline characteristics of per-protocol population. Supplemental Table S2. Overview of primary and secondary endpoints per-protocol. Supplemental Table S3. Subgroup analyses per-protocol for the primary endpoint mortality. Supplemental Table S4. Subgroup analyses per-protocol for the secondary endpoint recurrence. Supplemental Table S5. Subgroup analyses per-protocol for the secondary endpoint secondary infection. Supplemental Table S6. Overview of disease activity scores of intention-to-treat population. Supplemental Figure F1. Trial flow chart per-protocol. Supplemental Figure F2. Kaplan-Meier-curve of time to death per-protocol. [file 12890_2023_2794_MOESM1_ESM.pdf]

SUPPORTING INFORMATION TO

ADJUNCT PREDNISONE IN COMMUNITY-ACQUIRED PNEUMONIA: 180-DAY OUTCOME OF A MULTICENTRE, DOUBLE-BLIND, RANDOMIZED, PLACEBO-CONTROLLED TRIAL"

Supplemental table S1. **Baseline characteristics of per-protocol population**

Supplemental table S2. **Overview of primary and secondary endpoints per-protocol**

Supplemental table S3. **Subgroup analyses per-protocol for the primary endpoint mortality**

Supplemental table S4. **Subgroup analyses per-protocol for the secondary endpoint recurrence**

Supplemental table S5. **Subgroup analyses per-protocol for the secondary endpoint secondary infection**

Supplemental table S6. **Overview of disease activity scores of intention-to-treat population**

Supplemental figure F1. **Trial flow chart per-protocol**

Supplemental figure F2. **Kaplan-Meier-curve of time to death per-protocol**

Supplemental table S1. Baseline characteristics of per-protocol population

	Placebo (n=359)	Prednisone (n=355)	p-value
**General characteristics**			
Age, median (IQR), y	72 (60, 82)	74 (61, 84)	0.19
Male Gender, No. (%)	225 (62.7%)	217 (61.1%)	0.67
**Clinical variables**			
Days with symptoms, median (IQR), d	4 (2, 7)	4 (2, 7)	0.92
Temperature, median (IQR), °C	37.6 (37, 38.3)	37.5 (37, 38.2)	0.75
Systolic blood pressure, median (IQR), mmHg	123.5 (110, 140)	124 (110, 140)	0.73
Heart rate, median (IQR), bpm	82 (71.5, 96)	84 (74, 94)	0.74
Respiratory Rate, median (IQR), bpm	20 (18, 24)	20 (18, 24)	0.79
SaO2, median (IQR), %	94 (92, 96)	95 (92, 96)	0.94
Confusion, No. (%)	25 (7.0%)	21 (5.9%)	0.57
**Laboratory values**			
Procalcitonin, median (IQR), ng/mL	0.4285 (0.168, 2.3)	0.46 (0.17, 2.58)	0.97
C-reactive protein, median (IQR), mg/L	161.5 (73.9, 249.6)	159 (80.1, 245.5)	0.76
Leukocytes, median (IQR), x10^9/L	11.96 (8.8, 15.6)	12.09 (8.8, 15.58)	0.72
**PSI score†**			
PSI class I, No. (%)	41 (11.4%)	43 (12.1%)	0.38
PSI class II, No. (%)	66 (18.4%)	65 (18.3%)	
PSI class III, No. (%)	84 (23.4%)	62 (17.5%)	
PSI class IV, No. (%)	122 (34.0%)	135 (38.0%)	
PSI class V, No. (%)	46 (12.8%)	50 (14.1%)	
Total PSI score, median (IQR) ,points	86 (65, 110)	93 (63, 115)	0.23
**Comorbidities**			
Diabetes mellitus, No. (%)	71 (19.8%)	64 (18.0%)	0.55
Insulin treatment, No. (%)	23 (32%)	21 (33%)	0.91
Chronic obstructive pulmonary disease, No. (%)	52 (14.5%)	70 (19.7%)	0.063
Heart failure, No. (%)	57 (15.9%)	72 (20.3%)	0.13
Cerebrovascular disease, No. (%)	29 (8.1%)	32 (9.0%)	0.66
Renal insufficiency, No. (%)	114 (31.8%)	114 (32.1%)	0.94
Neoplasia, No. (%)	21 (5.9%)	27 (7.6%)	0.35
Liver disease, No. (%)	11 (3.1%)	17 (4.8%)	0.24
Antibiotic pretreatment, No. (%)	87 (24.2%)	73 (20.6%)	0.24

Data are median (IQR) or number (%), unless otherwise stated. SaO2=saturation of oxygen. PSI=pneumonia severity index.

†The PSI is a clinical prediction rule to calculate the probability of morbidity and mortality in patients with community-acquired pneumonia; PSI risk class I corresponds to age ≤50 years, and no risk factors (≤50 points), risk class II to <70 points, risk class III to 71-90 points, risk class IV to 91-130 points, and risk class V to >130 points.(1)

Supplemental table S2. Overview of primary and secondary endpoints per-protocol

Endpoints	Placebo (n=359)	Prednisone (n=355)	Adjusted HR or OR (95%CI)*	P value
**Primary endpoint**				
Death from any cause – no. (%)	25 (7.0%)	35 (9.9%)	HR 1.13 (0.67 - 1.92)	0.645
**Secondary endpoints**				
CAP-related Death	7 (1.9%)	6 (1.7%)	OR 0.74 (0.24 – 2.30)	0.605
Re-Hospitalization – no. (%)	55 (15.3%)	67 (18.9%)	OR 1.24 (0.84 - 1.85)	0.279
Recurrent pneumonia – no. (%)	12 (3.3%)	29 (8.2%)	OR 2.56 (1.28 - 5.11)	0.008
Secondary infections	34 (9.5%)	61 (17.2%)	OR 1.97 (1.26 - 3.08)	0.003
Type of infection				
- dermatological	1 (3%)	5 (8%)		
- urogenital	10 (30%)	8 (14%)		
- pulmonary	11 (33%)	18 (31%)		
- intestinal	10 (30%)	22 (37%)		
- endocardium or foreign body	1 (3%)	4 (7%)		
- both urogenital and pulmonary	0 (0%)	2 (3%)		
New hypertension at day 180	6 (1.7%)	10 (2.8%)	OR 1.71 (0.61 - 4.76)	0.304
New insulin dependence at day 180	1 (0.3%)	8 (2.3%)	OR 7.62 (0.94 - 61.63)	0.057

Data are number (%) unless otherwise stated. HR=hazard ratio. OR=odds ratio. CI=confidence interval. CAP=community-acquired pneumonia.

*Adjusted for PSI[#] and age

[#]PSI=pneumonia severity index. The PSI is a clinical prediction rule to calculate the probability of morbidity and mortality in patients with community-acquired pneumonia; PSI risk class I corresponds to age ≤50 years, and no risk factors (≤50 points), risk class II to <70 points, risk class III to 71-90 points, risk class IV to 91-130 points, and risk class V to >130 points.(1)

Supplemental table S3. Subgroup analyses per-protocol for mortality

**Subgroup variables**	**Placebo (n=359)**	**Prednisone (n=355)**	**Cox regression, HR (95%CI)**	**p for heterogeneity (interaction)†**
**Median age**				0.350
Age ≤ 73 years	8/191 (4.2%)	6/174 (3.4%)	0.99 (0.33 - 2.94)	
Age > 73 years	17/168 (10.1%)	29/181 (16.0%)	1.33 (0.72 - 2.44)	
Initial median CRP				0.617
CRP ≤ 159 mg/L	14/178 (7.9%)	20/177 (11.3%)	1.38 (0.68 - 2.78)	
CRP > 159 mg/L	11/178 (6.2%)	14/176 (8.0%)	1.03 (0.46 - 2.34)	
**History of Chronic Obstructive Pulmonary Disease**				0.962
no	21/307 (6.8%)	25/285 (8.8%)	1.25 (0.69 - 2.26)	
yes	4/52 (8%)	10/70 (14%)	1.44 (0.42 - 4.90)	
**Pneumonia severity index‡**				0.601
PSI class I-III	4/191 (2.1%)	6/170 (3.5%)	1.81 (0.51 - 6.42)	
PSI class IV-V	21/168 (12.5%)	29/185 (15.7%)	1.12 (0.63 - 1.98)	
**Blood culture positivity**				0.470
Blood culture negative	22/315 (7.0%)	30/320 (9.4%)	1.10 (0.62 - 1.93)	
Blood culture positive	3/44 (7%)	5/35 (14%)	1.18 (0.24 - 5.73)	

Data are number (%) unless otherwise stated. HR=hazard ratio. CI=confidence interval.

‡The PSI is a clinical prediction rule to calculate the probability of morbidity and mortality in patients with community-acquired pneumonia; PSI risk class I corresponds to age ≤50 years, and no risk factors (≤50 points), risk class II to <70 points, risk class III to 71-90 points, risk class IV to 91-130 points, and risk class V to >130 points.(1)

†Cox proportional hazards model including an interaction term of the respective subgroup variable with treatment group.

Supplemental table S4. Subgroup analyses per-protocol for the secondary endpoint recurrence

Subgroup variables	Placebo (n=359)	Prednisone (n=355)	Logistic regression, OR (95%CI)	p for heterogeneity (interaction)†
**Median age**				0.525
Age ≤ 73 years	8/191 (4.2%)	15/174 (8.6%)	2.25 (0.92 - 5.48)	
Age > 73 years	4/168 (2.4%)	14/181 (7.7%)	3.43 (1.10 - 10.69)	
**Initial median CRP**				0.248
CRP ≤ 159 mg/L	3/178 (1.7%)	13/177 (7.3%)	4.52 (1.26 - 16.18)	
CRP > 159 mg/L	9/178 (5.1%)	16/176 (9.1%)	1.88 (0.81 - 4.39)	
**History of Chronic Obstructive Pulmonary Disease**				0.964
no	9/307 (2.9%)	20/285 (7.0%)	2.50 (1.12 - 5.59)	
yes	3/52 (6%)	9/70 (13%)	2.47 (0.61 – 10.02)	
**Pneumonia severity index‡**				0.832
PSI class I-III	6/191 (3.1%)	14/170 (8.2%)	2.79 (1.05 - 7.44)	
PSI class IV-V	6/168 (3.6%)	15/185 (8.1%)	2.51 (0.94 - 6.67)	
**Blood culture positivity**				0.015
Blood culture negative	7/315 (2.2%)	28/320 (8.8%)	4.21 (1.81 - 9.79)	
Blood culture positive	5/44 (11%)	1/35 (3%)	0.14 (0.01 - 1.77)	

Data are number (%) unless otherwise stated. OR=odds ratio. CI=confidence interval.

‡The PSI is a clinical prediction rule to calculate the probability of morbidity and mortality in patients with community-acquired pneumonia; PSI risk class I corresponds to age ≤50 years, and no risk factors (≤50 points), risk class II to <70 points, risk class III to 71-90 points, risk class IV to 91-130 points, and risk class V to >130 points. (1)

†Logistic regression including an interaction term of the respective subgroup variable with treatment group.

Supplemental table S5. Subgroup analyses per-protocol for the secondary endpoint secondary infection

Subgroup variables	Placebo (n=359)	Prednisone (n=355)	Logistic regression, OR (95%CI)	p for heterogeneity (interaction)†
**Median age**				0.405
Age ≤ 73 years	14/191 (7.3%)	28/174 (16.1%)	2.44 (1.24 - 4.82)	
Age > 73 years	20/168 (11.9%)	33/181 (18.2%)	1.68 (0.92 - 3.07)	
**Initial median CRP**				0.099
CRP ≤ 159 mg/L	17/178 (9.6%)	40/177 (22.6%)	2.76 (1.50 - 5.10)	
CRP > 159 mg/L	17/178 (9.6%)	21/176 (11.9%)	1.27 (0.65 - 2.51)	
**History of Chronic Obstructive Pulmonary Disease**				0.282
no	29/307 (9.4%)	43/285 (15.1%)	1.70 (1.03 - 2.81)	
yes	5/52 (10%)	18/70 (26%)	3.11 (1.05 - 9.19)	
**Pneumonia severity index‡**				0.505
PSI class I-III	15/191 (7.9%)	28/170 (16.5%)	2.33 (1.20 - 4.53)	
PSI class IV-V	19/168 (11.3%)	33/185 (17.8%)	1.69 (0.92 - 3.11)	
**Blood culture positivity**				0.673
Blood culture negative	28/315 (8.9%)	51/320 (15.9%)	1.94 (1.19 - 3.17)	
Blood culture positive	6/44 (14%)	10/35 (29%)	2.20 (0.69 - 7.07)	

Data are number (%) unless otherwise stated. OR=odds ratio. CI=confidence interval.

‡The PSI is a clinical prediction rule to calculate the probability of morbidity and mortality in patients with community-acquired pneumonia; PSI risk class I corresponds to age ≤50 years, and no risk factors (≤50 points), risk class II to <70 points, risk class III to 71-90 points, risk class IV to 91-130 points, and risk class V to >130 points. (1)

†Logistic regression including an interaction term of the respective subgroup variable with treatment group.

Supplemental Table S6. Overview of disease activity scores of intention-to-treat population

**CAP score***	**Placebo (n=366)**	**Prednisone (n=361)**	**p - value**
CAP score* at day 5, median (IQR) points	58.3 (40.2 - 74.0)	57.7 (40.8 - 76.3)	0.526
CAP score* at day 30, median (IQR), points	84.3 (71.9 - 88.8)	81.1 (67.5 - 88.2)	0.095
CAP score* at day 180, median (IQR), points	88.2 (80.5 - 94.1)	88.2 (78.1 - 94.1)	0.171

Data are median (IQR) unless otherwise stated. CAP = Community-acquired Pneumonia.

*The CAP score is a disease-specific activity score for CAP. It ranges from 0 to 100, 0 marking the worst, 100 the best score.(2)

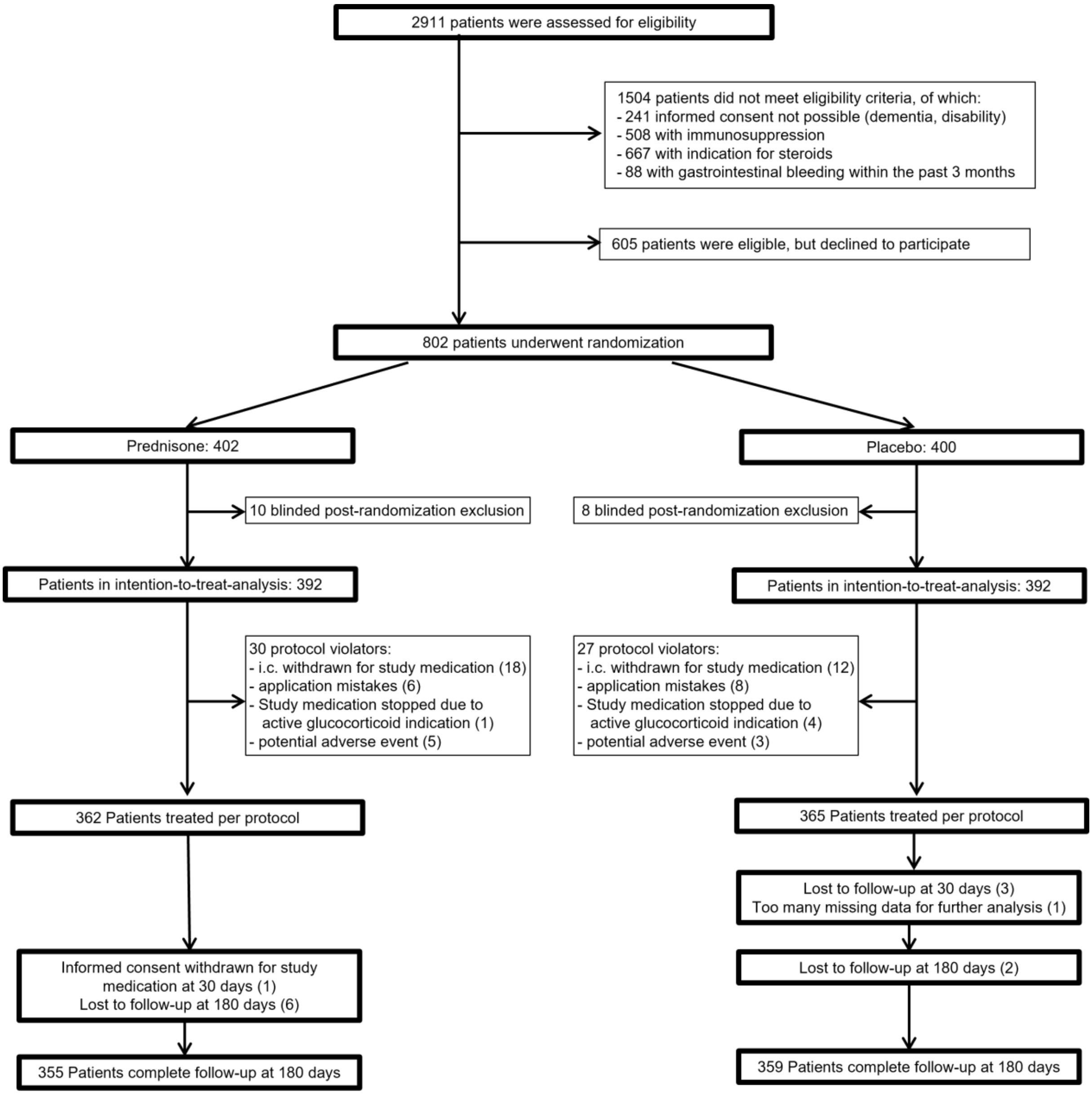

Supplemental figure F1. Trial flow chart per-protocol

The difference between study flow chart of STEP analysis 30 days and 180 days is due to new information and change of diagnosis during hospitalisation (one patient suffered of diverticulitis instead of pneumonia).

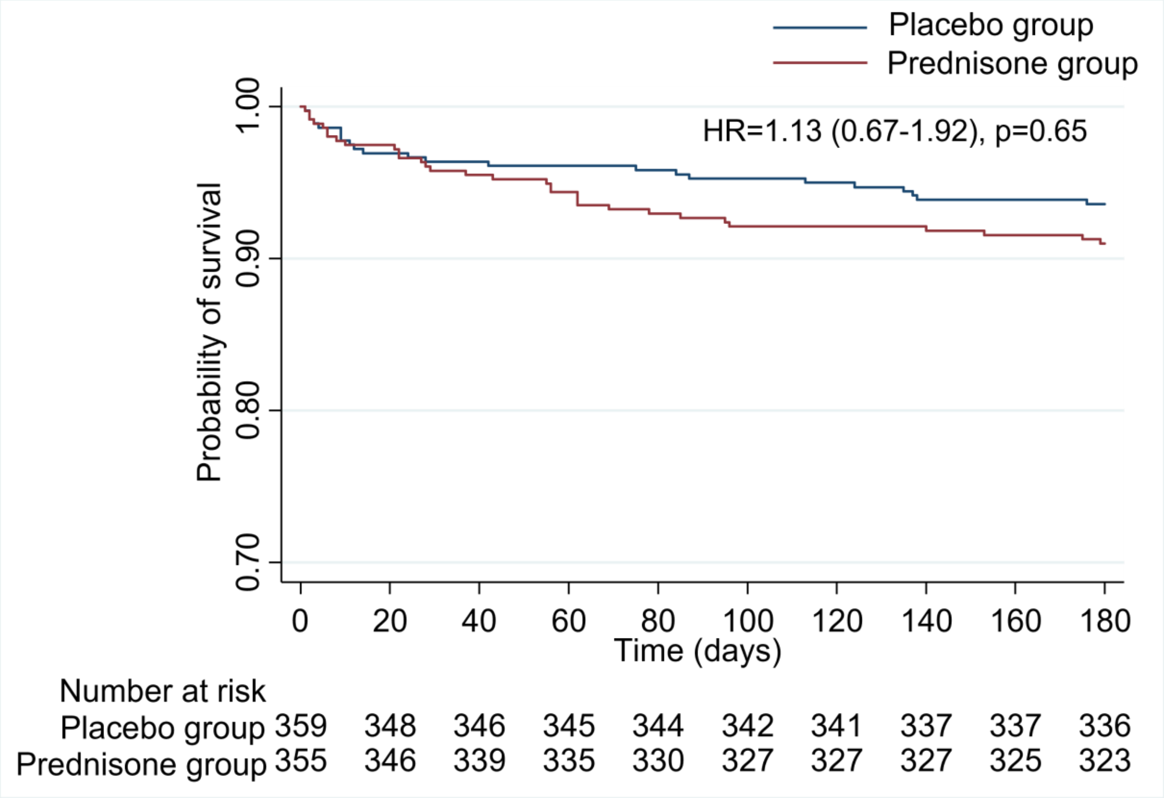

Supplemental figure F2. Kaplan-Meier-curve of time to death per-protocol

REFERENCES

1. Fine MJ, Auble TE, Yealy DM, Hanusa BH, Weissfeld LA, Singer DE, et al. A prediction rule to identify low-risk patients with community-acquired pneumonia. N Engl J Med. 1997;336(4):243-50.
2. El Moussaoui R, Opmeer BC, Bossuyt PM, Speelman P, de Borgie CA, Prins JM. Development and validation of a short questionnaire in community acquired pneumonia. Thorax. 2004;59(7):591-5.
